# An Individualized Machine Learning Approach for Human Body Weight Estimation Using Smart Shoe Insoles

**DOI:** 10.3390/s23177418

**Published:** 2023-08-25

**Authors:** Foram Sanghavi, Obafemi Jinadu, Victor Oludare, Karen Panetta, Landry Kezebou, Susan B. Roberts

**Affiliations:** 1 Department of Electrical and Computer Engineering, Tufts University, Medford, MA 02155, USA; obafemi.jinadu@tufts.edu (O.J.); karen@ece.tufts.edu (K.P.);; 2 Friedman School of Nutrition Science and Policy, Tufts University, Medford, MA 02155, USA; susan.roberts@tufts.edu

**Keywords:** human body weight estimation, smart shoe insoles, machine learning, predictive modeling

## Abstract

Rapid significant weight fluctuations can indicate severe health conditions such as edema due to congestive heart failure or severe dehydration that could require prompt intervention. Daily body weighing does not accurately represent the patient’s body weight fluctuations occurring within a day. The patient’s lack of compliance with tracking their weight measurements is also a predominant issue. Using shoe insole sensors embedded into footwear could achieve accurate real-time monitoring systems for estimating continuous body weight changes. Here, the machine learning models’ predictive capabilities for continuous real-time weight estimation using the insole data are presented. The lack of availability of public datasets to feed these models is also addressed by introducing two novel datasets. The proposed framework is designed to adapt to the patient, considering several unique factors such as shoe type, posture, foot shape, and gait pattern. The proposed framework estimates the mean absolute percentage error of 0.61% and 0.74% and the MAE of 1.009 lbs. and 1.154 lbs. for the less controlled and more controlled experimental settings, respectively. This will help researchers utilize machine learning techniques for more accurate real-time continuous weight estimation using sensor data and enable more reliable aging-in-place monitoring and telehealth.

## 1. Introduction

Extreme weight fluctuations can indicate severe health conditions causing edema or dehydration, as well as dietary and environmental factors influencing body fluid. Peripheral edema (edema of the lower limbs) is particularly common in older people. Besharat et al. [1] reported a 19% to 20% weighted prevalence of edema in older adults in the U.S. between 2000 and 2016. Deficiencies in the venous or lymphatic systems, congestive heart failure, and certain medications are some of the causes of peripheral edema [1]**.** Other factors that can cause edema include health conditions such as obesity or a history of deep vein thrombosis, a sedentary lifestyle, or jobs requiring long hours of standing [2,3]. There is no cure for peripheral edema, but it can be managed by taking diuretic medications; however, rapid monitoring of alterations in health or environmental contributors is necessary. Similarly, dehydration is prevalent in older adults [4]**,** and without active monitoring, this can lead to acute health crises including death.

Daily body weighing requires the individual to step on the scale and does not provide enough information regarding the weight fluctuations that occur in the edematous patients’ body and in individuals at risk of dehydration throughout the day. Additionally, the patient’s lack of compliance with tracking their weight measurements on their own, despite this being prescribed by their healthcare provider, is a predominant issue. Using embedded sensors in their footwear could help mitigate these issues. Roberts et al. [5] summarized findings that indicate that a healthy weight and nutrition maintenance are vital to healthy aging, which can be achieved by continuous real-time weight tracking. Thus, a real-time weight monitoring system can help not only the healthcare professionals, but also family members, to keep track of the patient’s body weight fluctuations, alert them when a drastic change is recorded, and provide timely interventions should an emergency arise.

Recently, incorporating artificial intelligence (AI) in real-time health monitoring has become a particularly important tool in maintaining and tracking good health. The data collected from wearable devices such as smartwatches and shoe insole sensors provide valuable insights and can help in training the AI models for different tasks. While significant advancements have been made in monitoring several health vitals such as heart rate, oxygen levels, and sleep patterns, real-time continuous weight monitoring is yet to reach such levels of advancement.

The challenge of body weight estimation using shoe insole sensors has recently gained popularity in the research community. In 2018, Tan et al. [6] used the Pedar-X sensor for data collection from a single participant for six distinct activities over time, including limping, walking at low, medium, and fast speeds, and running. The authors further proposed a mathematical model for estimating weight by measuring the average force applied to all pressure sensors from both legs and computing the mean over a finite period. The authors reported a 6% to 10% error in body weight estimation.

Muzzafar et al. [7] posited that the existing shoe insoles fail to account for the effect of the ground surface in the sensor layout pattern, thus often incurring a high error rate in measurements retrieved from essential pressure points. To alleviate this, Muzzafar et al. [7] proposed a novel ergonomic shoe insole design that involves sandwiching several sensors in a three-layered pattern for optimized and more robust force measurements.

Moon et al. [8] investigated the negative impact of postural instability and fatigue on the sensor data, which increased the error rate in weight estimation modeling. The experimental simulations suggested that the retrieved data accuracy from shoe insole pressure sensors significantly improved by introducing stochastic resonance. They proposed a sub-sensory insole vibration mechanism to reduce the effect of fatigue and postural instability and restore the central area of pressure for more accurate force measurements. Velardo et al. [9] explored visual cues to study the feasibility of weight estimation from anthropometric features related to human appearance and correlated them to weight using a multiple-regression-analysis-based model.

Kim et al. [10] proposed a modified MobileNetV2 architecture-based multi-tasking deep learning model for classifying human activities and estimating speed and body weight using the shoe insole sensors of 72 participants. The inputs given to the model comprise the signal data, such as acceleration and pressure, extracted from the insole sensors. The paper records ~97% accuracy in activity classification and 6.85 kg mean absolute error for body weight estimation for the train-test split of 70% and 30%, respectively.

Thus, the existing weight estimation methods using shoe insole pressure sensors are plagued with several challenges that limit their usefulness in real-life applications. These include (a) high error rates and standard deviation, (b) poor experimental setups and data collection strategies, and (c) non-robust data analysis and evaluation methodologies.

**Figure 1:**
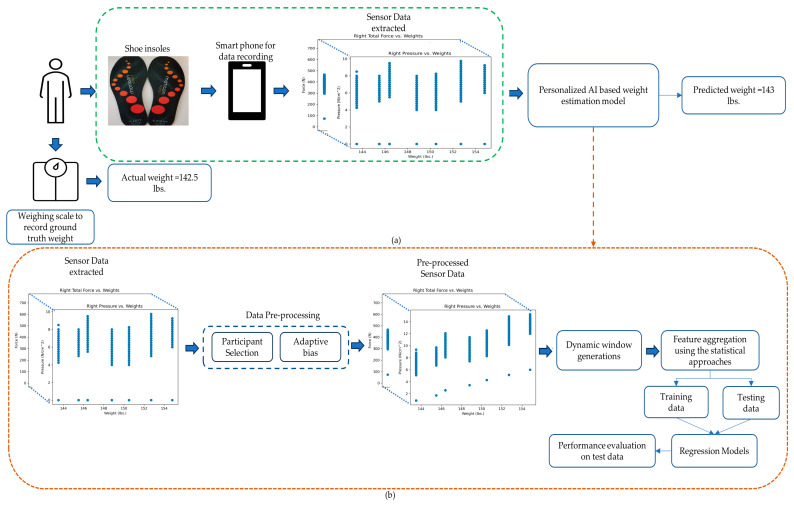
(**a**) Schematic pipeline of the weight estimation system with data collection (highlighted in green box) and (**b**) Detailed workflow of the Personalized AI based weight estimation model.

To address these challenges, this work introduces a more robust data collection, analysis, and evaluation methodology. The paper presents a comprehensive dataset, with 60 participants performing numerous activities in controlled and uncontrolled settings. Furthermore, every individual has a unique posture and gait pattern, generating unique sensor data. Moreover, two individuals with different weights can have similar feature ranges, thus requiring an individual specific model. Thus, this article also explores an artificial-intelligence-based solution to real-time weight monitoring using shoe insole sensors and a personalized machine learning algorithm. The following are the contributions of this paper:

(1) A novel experimental dataset for real-time continuous weight monitoring which includes measurements taken from participants performing actions such as sitting, standing, and walking.
(2) A novel automated adaptive bias computation to correct the error incurred in the sensor reading due to sensor initialization, improper flooring, shoe type, and posture.
(3) A novel dynamic windowing approach based on temporal dependencies for feature extraction.
(4) An individualized machine learning approach adapted to automatically adjust the prediction mechanism by self-retraining, which enables it to understand data patterns specific to individuals based on their postures, pressure points, and stability (Figure 1).

The remainder of this paper is organized as follows: Section 2 describes the data acquisition strategy. Section 3 describes the proposed methodology adopted to solve this problem. Section 4 describes the feature extraction implementation and results. Section 5 discusses key takeaways. Section 6 provides detailed instructions on how the data can be accessed. Section 7 concludes this study and discusses the future direction of research.

## 2. Data Acquisition

### 2.1. Sensor Information

In this paper, the data collection was performed using a commercially available Moticon shoe insole [11]. This shoe insole is currently being used for generating foot pressure maps for medical purposes and athletics. It comes in nine different sizes, covering 98% of adult sizes, and is usable for any shoe type [11]. Each shoe insole is fitted with sixteen pressure sensors, three acceleration sensors, and three angular rate sensors. It also has a portable battery and a Bluetooth connection for transmitting data between a computer and a mobile phone. Thus, the data acquired from each insole includes (i) a time stamp dependent on the sampling rate (s), (ii) the pressure values from all sixteen sensors (N/cm^2^), (iii) acceleration (g) and angular rates (dps) along the x, y, and z axes, (iv) the computed center of pressure (insole length/width) in the x and y axes, and (v) the total force (N). Thus, a total of 51 features are measured. More details regarding the pressure sensors’ functionality and data accuracy can be found in [12].

### 2.2. Experimental Setup

**Figure 2:**
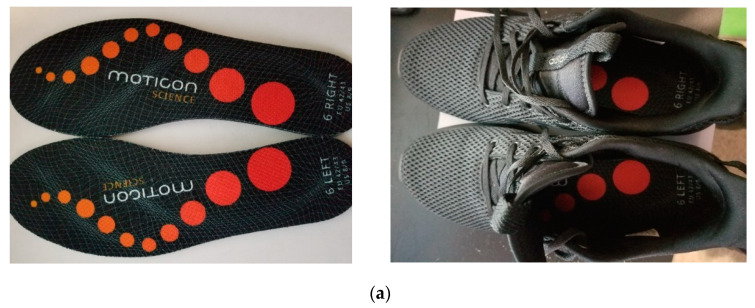

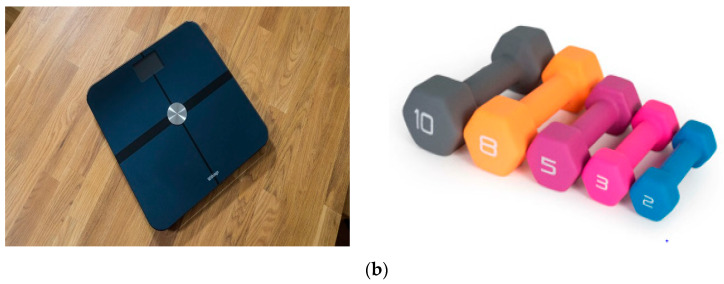
Shows images of (**a**) the Moticon shoe insoles and the embedded system of the shoe insoles in the shoes and (**b**) the Withings weighing scale and dumbbell weights for different loads carried by the participant [13]**.**

The data acquisition was performed following Institutional Review Board permission using two experimental setups of less controlled and more controlled environment settings. Both setups consisted of self-identifying male and female participants. In each setup, the participants were asked to insert shoe insoles of the appropriate size in their footwear, and the initial sensor calibration was performed. The participant’s ground truth weight was first recorded using the Withings scale. After this, the participants, depending on the experimental setup, were made to perform different activities with and without dumbbells. Figure 2 shows the experimental design for both setups, with illustrations of the shoe insole sensor and how it is fitted in a shoe, the Withings weighing scale, and the different dumbbell weights used.

The sensor data were then collected for each activity from the shoe insole sensors along with ground truth weight in pounds (lbs.), yielding more robust and less noisy data. The data recording and calibration were performed using a smartphone installed with the OpenGo software. The same software was used on a Windows laptop to convert the ‘.go ‘ files to ‘.txt’ files, which were forwarded to the proposed machine learning framework. Prior to the data acquisition, the Institutional Review Board permission was obtained from the Tufts University Health Sciences IRB.

(a) Experimental Setup 1: In the first experimental setup, the data were collected from 53 participants in a less controlled environment of varying standing surfaces (such as wooden, tiled, or concrete) and with different shoes worn by the participants (namely, heels, athletics shoes, or flip-flops). Each participant was then made to perform different tasks, such as standing, walking, and sitting, with and without graded series of dumbbell weights of up to 12 lbs., taking a total of 45 min per participant. The participants in this setup are between the ages of 18 and 70 and the data were acquired for each participant in one or two experimental iterations, with sensor calibration performed at the start of every iteration. The data here was collected at the sampling rate of 25 Hz.

(b) Experimental Setup 2: In this setup, the data were collected in a more controlled environment from seven participants in the age group of 20 to 32 years. Herein, each participant was asked to perform only the standing task with and without carrying dumbbells. The series of dumbbell weights carried by the participants here are 2, 3, 5, 7, 9, 11, and 13 lbs. To prevent reading fluctuations due to surface changes and shoe types, each participant wore athletic shoes, and the data were recorded while standing on the Withings scale (smart weighing scale). Furthermore, the data were acquired for each participant in three experimental iterations, with sensor calibration performed at the start of every iteration. The data here were collected at the sampling rate of 50 Hz. Table 1 shows the data distribution for participants in both experimental setups.

## 3. Proposed Methodology

### 3.1. Data Pre-Processing

Before implementing the machine learning algorithm, it is crucial to understand the data and pre-process the data to eliminate any noise present. Herein, the data pre-processing is categorized into two steps: (a) participant selection and (b) automated adaptive bias. 

**Figure 3:**
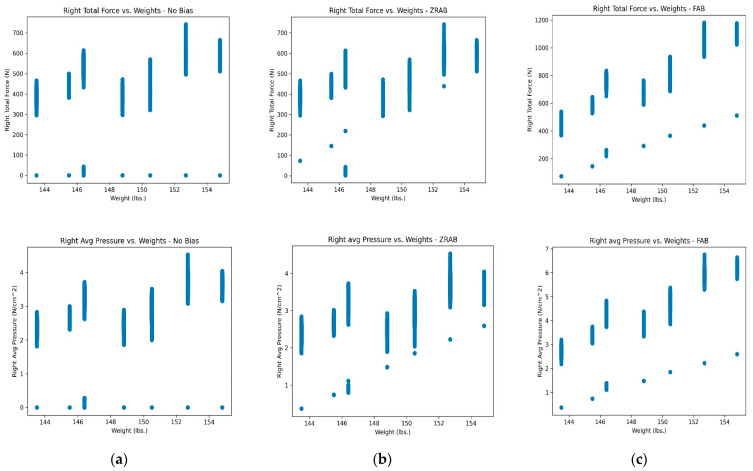
Comparing the sensor data before and after adding adaptive bias to them: (**a**) scatter plot of right total force vs. weight (top) and right avg pressure vs. weight (bottom), (**b**) scatter plot obtained after adding the adaptive bias to just zero-feature readings (ZRAB), and (**c**) scatter plot obtained after adding the adaptive bias to just all-feature readings (FAB).

(a) Participant selection: For the implementation of the proposed machine learning approach, it is essential to ensure that each participant should have at least two experimental iteration readings, so that at least one iteration datum can be used for training and validating the model and at least one iteration datum can be used for testing the model. On observing the data of each participant from both experimental setups, it was found that for setup 1, 37 participants out of 53 had data from two experimental iterations without any missing information, whereas for setup 2, all 7 participants had data from three experimental iterations. Thus, in this paper, for experimental setups 1 and 2, the analysis was performed on 37 and 7 participants, respectively. 

(b) Automated adaptive bias**:** For many participants, it was observed that several features extracted from the shoe insole sensors had a certain amount of ‘0**′** values, as shown in Figure 3a. These ‘0**′** values could be present across all weight experiments or in just a few experiments. These values could be due to the sensors getting initialized or participants not applying pressure and forces on some sensors due to improper posture or shoe type. However, if the uncorrected features are fed to the predictive machine learning model, it can lead to poor weight prediction performance as the model could fail to predict the correct weight, especially for low-range or near ‘0**′** values. This prompts a need to add a correction factor, a bias, to the sensor readings to offset these values. To accomplish this, an automated adaptive-bias-addition-based data pre-processing method is proposed in this paper. To automatically compute the bias value, a non-zero minimum feature value of the maximum weight in the weight class and the length of weight class are required from the sensor data. The following is the formula for computing the bias (B):

(1) $$B_{j}^{i}=\frac{\min\left(S_{j}>0\right)|max(Wc)}{L_{Wc}}*i$$

where Wc is the weight class, $L_{Wc}$ is the weight class length, i is the index of the weight class such that i = 1, 2, …, $L_{Wc}$, j is the feature index such that j = 1, 2, …, 40, $S_{j}$is j^th^ sensor feature value, and $B_{j}^{i}$ is the bias computed for the i^th^ weight class and j^th^ sensor feature. The weight class length corresponds to the number of weight experiments performed by a participant starting from no weight. For example, in experimental setup 2, the weights present in the Wc are 0, 2, 3, 5, 7,9, 11, and 13 lbs.; thus, the weight class length is 8. The unit associated with bias depends on the feature it is computed on; that is, if the feature is a force, the unit is newtons (N), or if the feature is pressure, the unit is N/cm^2^.

Here, the bias value adapts automatically and independently to each feature and weight class for every participant. If there is no non-zero minimum in the maximum weight class, the non-zero minimum from the next highest weight is computed. This process continues until a non-zero minimum is achieved. The following are the main reasons for the bias to be adaptive and independent:

(i) To maintain a positive correlation between increasing weights and feature values, a higher bias value needs to be added to the feature with an increase in weight.
(ii) Each feature can have a different value range or scale.
(iii) Every individual has a unique gait pattern, leading to a unique force and pressure distribution and value range, thus requiring a unique correction value.

In this article, two different settings of additive bias are tested, namely (i) zero-reading adaptive bias (ZRAB) and (ii) full adaptive bias (FAB). The following is the formula for updating the sensor reading:

(2) $${S\prime }_{j}^{i}=S_{j}^{i}+B_{j}^{i}$$

where S’ and S are the updated and existing sensor feature. In the case of ZRAB, bias is added only when the features with ‘0**′** values are updated, whereas in case of FAB, the bias is added to all the features. The effect of ZRAB and FAB can be seen in Figure 3b and Figure 3c, respectively.

### 3.2. Dynamic-Windowing-Based Feature Extraction

Data size is observed to be varying for each participant per dumbbell weight due to the variation in the acquisition time required to capture each data point. This discrepancy in data size can result in a bias in the machine learning model trained for predicting body weight. To overcome this issue, a dynamic windowing approach is proposed in this paper for feature extraction. Sliding window approaches and their variants have been used for several outlier detection and prediction tasks [14,15,16,17,18,19,20]. However, the current adaptive windowing methods often fail to account for temporal dependencies in the data. The proposed dynamic windowing approach considers these dependencies to automatically calculate the overlap ratio between the windows for each weight reading. The following are the formulae for the proposed dynamic windowing:

(3) $${Win}_{new}=\frac{L_{data\_sequence}+{Olap}_{new\;}*{\;Win}_{init}}{L_{samples}}$$

(4) $${Win}_{init}=\frac{L_{data\_sequence}}{L_{samples}}$$

(5) $${Olap}_{new}={Olap}_{init\;}*{\;Win}_{init}$$

where, $L_{data\_sequence}a$nd $L_{samples}$correspond to the data sequence length and initial window length for a specific weight, respectively; ${Win}_{init}$ and ${Olap}_{init}$ correspond to the initial window size and overlap rate, and ${Win}_{new}$ and ${Olap}_{new}$ correspond to the new window size and overlap rate, respectively. The initial window length ($L_{samples})$ and overlap rate (${Olap}_{init}$) parameters are user-defined inputs. This approach of dynamic windowing serves the following advantages:

(i) Removes the need for empirically determining the optimal window size, which is not practical for real-world applications, and
(ii) Provides a reasonable performance trade-off over time which is needed for extensive parameter search.

After generating different windows using the proposed dynamic windowing, a statistical approach based on trimmed mean (TM) is used to represent the data points obtained from each window as a single value. Thus, for every feature extracted from the shoe insole sensors, (i) windows are generated using the dynamic windowing, and (ii) trimmed mean is computed from each window, using a specific alpha value, and stored as a feature vector.

To compute trimmed mean, the window data points are first sorted in either ascending or descending order, from which some data points are removed from both ends, and the arithmetic mean of the reaming values is then computed. This removes the extreme low and high points, where the outliers or noise are usually present. The number of data points being eliminated is controlled by an α value. The following are the formulae for establishing trimmed mean [21]:

(6) $$TM\left(\alpha \right)=\frac{1}{{Win}_{new}-2\;*\;k}\;*\;\sum_{j=k}^{{Win}_{new}-k}{data}_{j}$$

(7) $$k=\frac{\alpha \;*\;{Win}_{new}}{100}$$

where, α and k are the percentage and the number of data points to be removed, respectively. TM (α) is the trimmed mean value of the windowed sensor data. The value of k is computed using the user-defined α value in the range of 0–50. The trimmed mean serves the following advantages:

(i) It helps reduce the effect of outliers or noise, by eliminating those data points when computing the arithmetic mean, and
(ii) When α = 0 and 50%, the trimmed mean works as the arithmetic mean and median statistical approach.

## 4. Experiments and Results

The goal of this regression model is to generate human body weight predictions (y), given features (Φ) extracted from a smart shoe insole sensor. This can be formulated as:

(8) $$y=\sum_{n=0}^{N}c_{n}\phi_{n}=\Phi C^{T}$$

where C is an N-size row vector of learnable model weights and N corresponds to the feature size defined below:

(9) $$C=[c_{0},c_{1},c_{2},\ldots ,c_{N}],\;c_{0}\;\mathrm{is the bias term.}$$

Φ is the corresponding N-size row vector of features from the left and right shoe insoles:

(10) $$\Phi =[\phi_{0}=1,\phi_{1},\phi_{2},\ldots ,\phi_{N}]$$

### 4.1. Feature Extraction and Training

Since a positive correlation exists between the (weights and pressure) and (weights and total force), this paper utilizes these features as Φ in building the regression model to predict human body weight, y. Sixteen pressure features and one total force feature each from the right and left shoe insole are used to build the model. Additionally, six features are synthesized from these thirty-four features, including the sum and average of the pressure and total force features from the insoles individually and combined. Thus, forty features are used. For each insole reading, the following pre-processing steps were carried out:

(i) Addition of an adaptive bias to each insole reading to give more consistently correlated sensor readings.
(ii) Dynamic windowing is used to extract statistically aggregated feature vectors which form a feature matrix that serves as input to the regression model.

The feature matrix generated, along with the corresponding ground-truth labels (weights), is fed into the regression model for training and testing. Since every human has a unique gait pattern, the force and pressure distribution consequently differ. Hence, individual-specific prediction models are trained, meaning there is a unique model for each participant. A 5-fold cross-validation is applied to validate the performance of each machine learning model adopted. These machine learning models include ExtraTreeRegressor, Light Gradient Boosting (LightGBM), and Categorical Boosting (CatBoost) with ExtraTreeRegressor giving the best results; an open-source AutoML library, Pycaret [22], was used for model building.

### 4.2. Evaluation Metrics

The evaluation metrics considered for the regression-based task of weight estimation are the mean absolute error (MAE) [23], mean squared error (MSE) [24], root mean squared error (RMSE) [23], coefficient of determination (R^2^) [25], and mean absolute percentage error (MAPE) [26]. The formulae for each metric are given in Equations (11)– (16):

(11) $$MAE=\frac{1}{n}\sum_{i=1}^{n}\left|e_{i}\right|$$

(12) $$MSE=\frac{1}{n}\sum_{i=1}^{n}e_{i}^{2}$$

(13) $$RMSE=\sqrt{\frac{1}{n}\sum_{i=1}^{n}e_{i}^{2}}$$

(14) $$R^{2}=1-\frac{\sum_{I=1}^{n}e_{i}^{2}}{\sum_{I=1}^{n}{\left(y_{i}-\overset{-}{y}\right)}^{2}}$$

(15) $$MAPE=\frac{1}{n}\sum_{i=1}^{n}\left|\frac{e_{i}}{y_{i}}\right|$$

(16) $$e_{i}=y_{i}-\hat{y_{i}}$$

where, ($y_{i},\hat{y_{i}})$ is the ground truth weight, weight prediction pair, and $\overset{-}{y}$ is the mean of ground truth weights.

### 4.3. Experiments

Experimentations were implemented in two phases: (a) Phase 1: This was carried out on seven participants, with four self-identifying males and three self-identifying females. For each participant, at least three independent experiments were performed in a more controlled environment, where experiments 1 and 3 were used for training (specifically, 80% for training and 20% for validation), and experiment 2 was solely used for testing. The aim of experiments in this phase is to narrow down the optimal pipeline pre-processing steps which include steps to explore the effects of (i) adding adaptive biases to the sensor data, (ii) the length of samples used to compute the initial window size for dynamic windowing, and (iii) the type of aggregation method used, and parameter selection used to generate the feature matrix.

**Figure 4:**
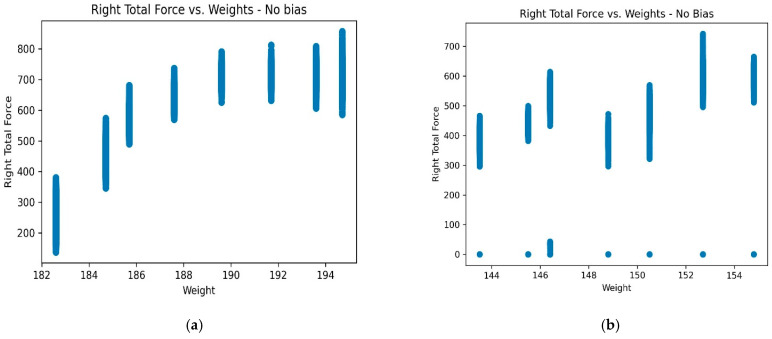
Compares the sensor readings of (**a**) participant 1 with relatively well-balanced force and pressure distribution, (**b**) participant 3 with unbalanced distributions prior to adding any form of adaptive bias. Participant 1 shows a more consistent correlation between right total force and weights while participant 3 shows an inconsistent correlation along with zero-readings, emphasizing the need for bias addition.

(i) Effect of adaptive bias addition: The effect when including the full adaptive bias (FAB) and the zero-reading adaptive bias (ZRAB) is explored on participants with a relatively well-balanced foot pressure distribution and participants with unbalanced foot pressure distribution (Figure 4). For this exploration, the aggregation method used to extract the feature matrix was the vanilla mean which corresponded to an alpha-trim mean with α = 0%. The results are shown in Table 2.

Table 2 captures the effects of incorporating an adaptive bias on balanced and unbalanced participant data. For participants with unbalanced data (participants 2–7), it can be observed that adding the FAB gives the best performance when compared to the ZRAB and when no bias is included. Comparing the ZRAB and no bias, the performance trend is inconsistent, with the ZRAB performing better for some participants and worse for others. This is because most participants have more non-zero infinitesimal readings than actual zero readings, and because adding the adaptive bias to exclusively the zero readings is insufficient, leading to mispredictions. Whereas the performance margin is larger for the FAB and no-bias cases, FAB generates predictions with an MAE approximately 1.72 lbs. lower, on average, than the no-bias case. However, for the participant with relatively well-balanced data, there is a smaller improvement margin between the FAB and the no-bias instances, with the FAB producing predictions with approximately 0.26 lbs. lower MAE than the no-bias case.

**Figure 5:**
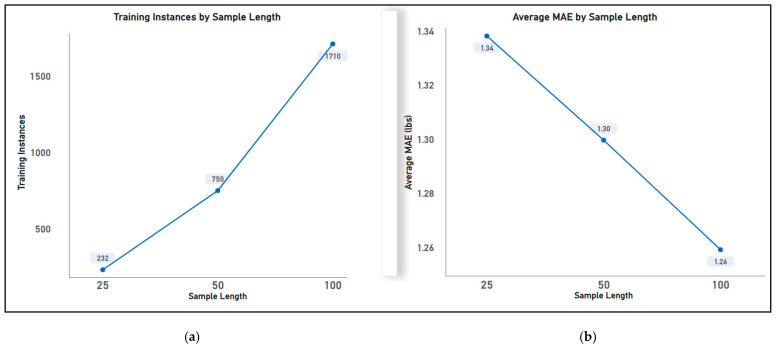
Illustration of effects of length of sample on (**a**) training instances, as the sample length increases, the number of training instances increases, and (**b**) model performance, as the length of samples increases, a model performance improves. Training instances and MAE values are averaged across all seven participants of experiments carried out in phase 1.

This shows that the effect of adding bias tends to diminish with a balanced dataset, which is expected since a balanced dataset has little to no zero readings. The pressure and force points are already largely positively correlated with the weights. Figure 4 shows the difference in the sensor readings of participants with balanced and unbalanced data, with no bias highlighting the degree of inconsistency in the correlation between weight and force. It is observed that for the balanced data, there is a reliable linear relationship between force and weight when compared to the unbalanced data, which suggests a lower dependence on the adaptive bias in the case of the balanced participant data and a higher dependence on the adaptive bias in the case of the unbalanced participant data, which aligns with the results captured in Table 2. Based on these results, the FAB was applied to all subsequent experiments.

(ii) Effect of the length of samples: The sample length () is used to compute the sizes of the initial and dynamic windows. In this paper, the following sample lengths of 25, 50, and 100 were explored to obtain corresponding window lengths, with an overlap ratio set to 20% (0.2). Similar to the previous experiment, the statistical aggregation method of vanilla mean is used here to extract features. Figure 5a shows that as the length of samples increases, the number of training instances increases. This is because with a smaller sample length, the initial and dynamic windows are larger (refer to Equations (3) and (4)), meaning more samples are extracted per window over which aggregation is performed to produce a single training instance. This generates less training instances. Conversely, with a larger sample length, smaller sizes of initial and dynamic windows are created, meaning fewer samples are extracted per window and aggregated over, producing a higher number of training instances. From Figure 5b, it can be observed that the MAE reduces with an increase in the sample length; this is because with smaller window sizes such as 25 and 50, more samples are extracted per window. This increases the likelihood of extracting noisy, uncorrelated samples that are less likely to reflect the true sensor estimates upon statistical aggregation. Therefore, the length of samples used for subsequent experiments was 100.

(iii) Effect of the Aggregation Method: Next, the effect of the aggregation approach used to extract the feature matrix from the dynamically windowed sensor readings was explored with the FAB and a sample length of 100. The results are captured in Table 3 above. Table 3 shows the effects of the aggregation strategies such as the mean, median, and alpha-trim mean with the FAB added. It can be observed that alpha-trim mean with α = 15% outperforms other statistical aggregation strategies for most participants. This is the case because a 15% trim factor is sufficient to trim outlier readings without eliminating useful positively correlated sensor readings. α below 15% tends to show slightly lower performance, as outlier readings are not adequately eliminated, while trim values above this also show a drop in performance because in most cases, useful sensor readings are also trimmed off along with outlier readings. Table 4 shows the finalized results for phase 1 experiments on all metrics considered.

Based on phase 1 experiments carried out to establish optimal pre-processing steps along with corresponding parameter selection, FAB addition, a sample length of 100, and an alpha-trim mean with α = 15% were selected and used for phase 2 experiments.

(b) Phase 2: This was carried out on 37 participants, with 22 self-identifying males and 15 self-identifying females. For each participant, exactly two independent experimental iterations were recorded in a less controlled experimental environment setup, where participants were allowed to wear a more diverse range of shoe options and with the experiments performed at different locations. Experimental iteration 1 was used for training (with an 80–20% split for training and validation, respectively), and experimental iteration 2 was used for testing. Experiments in this phase were primarily carried out to (i) evaluate phase 1 pipeline performance on a larger scale and (ii) mirror the performance on real-world deployment as closely as possible.

Table 5 shows results from experiments carried out in phase 2 on 37 participants consisting of 22 and 15 self-identifying males and females, respectively, using the pre-processing steps obtained from experiments in phase 1. It can be observed that the results are relatively consistent in both experimental phases. This shows that the proposed system is robust to environmental changes such as shoe types, posture, and flooring surfaces.

**Figure 6:**
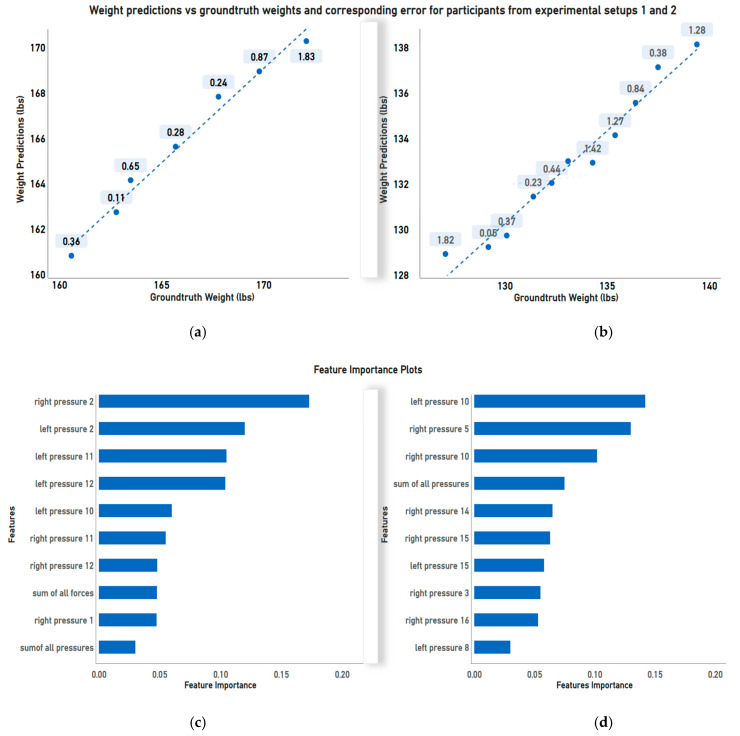
(**a**,**b**) Illustrate the scatter plots of the predicted weights vs. ground truth weights with a regression line and MAE per weight reading for participants in experiments in phases 1 and 2, respectively. (**c**,**d**) Illustrate the corresponding feature importance plots for the participants in (**a**,**b**).

Figure 6 presents the results of randomly selected Participant 2 (of 7) from phase 1 experiments and Participant 3 (of 37) from phase 2 experiments. Figure 6a,b show the scatter plot of predicted weight vs. the ground truth weights and their corresponding MAE for each weight class as a scatter point. Figure 6c,d display the corresponding feature importance plots for both participants. This shows how each participant generates different important features due to the varying foot pressure and force distribution resulting from their unique gait patterns, emphasizing the need for individual-specific prediction models for each participant.

Table 6 illustrates the performance analysis of the proposed method on both the environmental settings with respect to the existing state-of-the-art method. It can be observed that the proposed methodology achieves an MAE performance of nearly 1 lbs. compared to the MAE of 15.10 lbs. achieved by Kim et al. in [10]. This is because the proposed method generates a personalized model for every participant rather than using the whole dataset to train a single machine learning model as performed in [10].

## 5. Discussion

The study illustrates that using an individualized machine learning approach for body weight estimation significantly impacts the system’s prediction performance. It is essential to generate a predictive machine learning model for every participant’s data individually, because two individuals can exhibit similar force- or pressure-sensor-reading ranges, despite having different weight distributions (Figure 4). If a machine learning model is trained on the overall data, there is a high possibility that the model may fail to distinguish between the different weight classes and predict body weight with a large margin of error (MAE). The personalized approach helps mitigate this issue by generating models for every individual instead of training a single model on collective data. Results from Table 6 show that the proposed method yields a significantly lower MAE value compared to the deep-learning-based model [10], where the model is trained and tested on the entire dataset.

Experimental results indicate that adding an adaptive bias to sensor readings, using the appropriate windowing scheme and statistical aggregation method, plays a vital role in improving the system’s weight prediction performance. Results in Table 2 indicate that FAB yields a significantly improved performance. Compared to the model trained with no bias, FAB yields a 54.89% reduction in average MAE, and compared to the ZRAB model, there is a 52.05% reduction in average MAE across the seven participants in experimental setup 2. The reason is that in FAB, the adaptive bias addition ensures that a positive correlation is maintained between the force and pressure features with respect to weight, which ZRAB can fail to maintain when presented with near ‘0′ or low-range values. This positive correlation between the features and weight is extremely crucial for correct weight prediction.

Results in Figure 5 show that using a higher initial sample length for dynamic windowing produced lower MAE values and a higher number of training instances. However, the sample length hyperparameter needs to be tuned to an optimal value to ensure a good trade-off is achieved between the number of training instances and the number of datapoints in the window. This is because with the increase in the initial sample length, there is an increase in the number of training samples and a decrease in the number of data points in the window.

**Figure 7:**
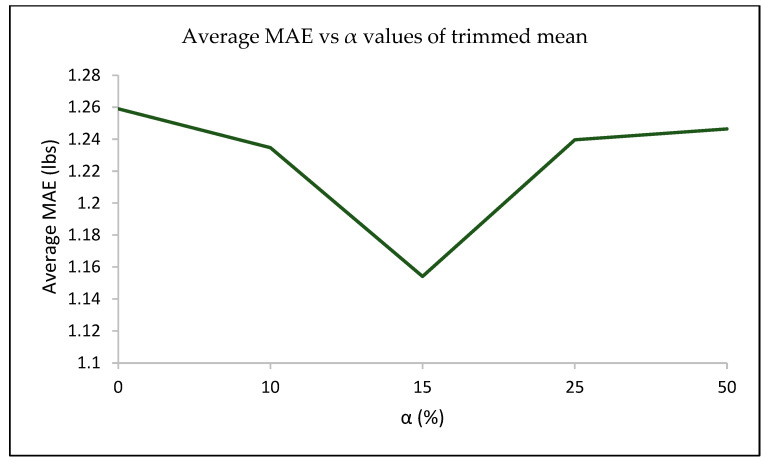
Compares the average MAE of seven participants (from experimental setup 2) across different α values, with α = 15% giving the lowest MAE on average.

Results in Table 3 suggest that using the α = 15% in the alpha-trimmed mean method for feature extraction generates the lowest averaged MAE value in experimental setup 2. It is essential to tune the hyperparameter value α to an optimal value to ensure that a trade-off between outlier removal and reliable data used for averaging over is achieved. From Figure 7, it can be observed that with an increase in α up to 15%, there is a decrease in the averaged MAE values; however, an opposite trend is noticed when α is increased beyond 15%. This is because lower α values are not sufficient to eliminate all outliers while higher α values tend to eliminate both noisy outliers and essential data required by the machine learning model.

Lastly, the study shows that the proposed individualized method of body weight estimation achieves comparable results in both controlled and less controlled environmental settings. This demonstrates the system’s robustness to an individual’s standing posture and environmental settings such as shoe type and flooring surfaces, which is important for real-world applicability.

## 6. How to Use the Dataset

**Figure 8:**
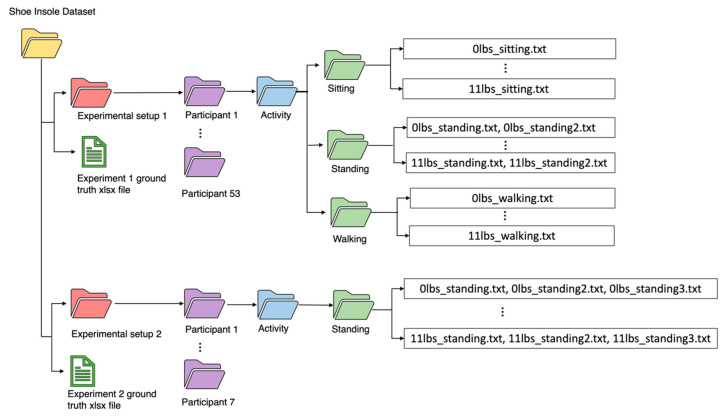
Directory structure and sensor measurement file-naming convention of the Shoe Insole Dataset.

The shoe insole dataset contains over 2200 shoe insole sensor measurements collected from 60 participants across experimental setups 1 and 2 which are each presented as subfolders in the database. Accompanied by these subfolders are two .xlsx files which correspond to the ground truth weights of participants in experimental setups 1 and 2. For experimental setup 1, the folder contains subfolders corresponding to each participant’s data, and each participant has an activity subfolder that contains the three activities considered; sitting, standing, and walking. For each of these three subfolders, the shoe insole sensor measurements are provided as text files. Experimental setup 2 has a similar structure. Each text file includes the following columns: pressure values(N/cm^2^), total force values (N), angular rate values along the x, y, and z-axis (dps), central pressure values for the left and right insole sensors, and time stamp(s). The naming convention of the sensor-reading text files is discussed in Section 6.1. Figure 8 shows the directory structure of the shoe insole dataset.

### 6.1. File Naming Convention

In the Shoe Insole Dataset, the text file names of the shoe insole measurements encode information about the experiments as shown in Figure 8. It consists of three fields; the first two fields are separated by an underscore, and the final field is appended at the end of the second field. For illustrative purposes, two names are considered, 0lbs_sitting.txt and 11lbs_standing2.txt.

1. Weight field: This corresponds to the weight of the dumbbell carried by the participant, where 0 lbs. is the participant’s base weight where no dumbbells are carried, and 11 lbs. means the participant carried dumbbells that weigh 11 lbs.
2. Activity field: This corresponds to the activity carried out by the participant during data capture. For experimental setup 1, it could be sitting, standing, or walking, while for experimental setup 2, the activity is strictly standing.
3. Iteration number field: This is the number that comes immediately after the activity field. When the iteration number is not included it implies iteration 1. For example, “0lbs_sitting.txt” is the first iteration of the participant, and 11lbs_standing**2**.txt is the second iteration for the 11 lbs. weight reading.

### 6.2. How to Access

Availability of the dataset is crucial for reproducibility and research transparency. The Shoe Insole Dataset is available on request for research purposes. For access, kindly contact the authors.

## 7. Conclusions

A personalized-learning-based approach for weight prediction that automatically adapts to data patterns specific to a person rather than the conventional generic-learning-based approach is presented. The method accounts for the unique postures, pressure points, and gait patterns of the individuals. A novel automated adaptive bias is also proposed to correct the insole sensor zero readings from participants that hamper the machine learning model performance. Subsequently, a novel dynamic windowing method adapting to the variation in data sample size is also proposed for feature extraction. For the aggregation of the extracted features, the alpha-trim mean approach was adopted. To help implement the proposed framework, this paper introduces two novel datasets for body weight estimation in real-time, using shoe insole sensors. Each dataset is acquired in different environmental settings, such as flooring surfaces and shoe types, with different activities. The mean absolute percentage error and the MAE recorded from the proposed methodology are 0.61% and 1.009 lbs., respectively, for the less controlled experiments with 37 participants and 0.74% and 1.154 lbs. for the more controlled experiments with 7 participants. This illustrates the proposed framework’s robustness on shoe types, flooring surfaces, and posture. Future efforts will be focused on (a) reducing the error margin between the weight estimations and ground truth by improving the data collection strategy and developing algorithms more robust to noise for better applicability in real-life scenarios, (b) developing an adaptive statistical aggregation mechanism that automatically selects the optimal aggregation method per participant rather than adopting the optimal method for most participants, (c) activity recognition such as standing, sitting, and walking, and (d) developing machine learning frameworks for weight estimation when the participant is in motion.

## Figures and Tables

**Table 1:** Data distribution in both experimental setups**.**

Experimental Setup	Activities Recorded	Number of Female Participants	Number of Male Participants
1	Standing, Walking, and Sitting	23	30
2	Standing	3	4

**Table 2:** Illustrates the effect of no bias, full adaptive bias (FAB), and zero-reading adaptive bias (ZRAB) using the vanilla mean aggregation. The FAB outperforms both other cases for all seven participants considered with unbalanced data, and for the participant with balanced data, the effect of adding the bias diminished. The results in bold indicate the best results observed for each participant.

MAE (lbs.) ↓							
	Balanced Data	Unbalanced Data					
**Method\Participant #**	1	2	3	4	5	6	7
No Bias	2.011	2.090	4.040	4.239	2.027	2.061	2.931
ZRAB	2.046	0.840	4.626	4.036	1.928	1.942	2.834
FAB	**1.753**	**0.637**	**2.798**	**0.597**	**1.015**	**1.433**	**0.580**

Upward arrow (↑) means higher values correspond to better performance. Downward arrow (↓) means lower values are correspond to better performance, which holds for all the tables reported.

**Table 3:** Comparison between different aggregation methods in feature matrix extraction using FAB and sample length of 100. The alpha-trim mean with α = 15% outperforms the other aggregation methods for most participants. The results in bold indicate the best results observed for each participant. α

MAE (lbs.) ↓								
		Balanced Data	Unbalanced Data					
**Aggregation\Participant #**		1	2	3	4	5	6	7
Mean	α = 0%	1.753	0.637	2.798	0.597	1.015	1.433	**0.580**
Median	α = 50%	**1.678**	**0.579**	2.759	0.564	1.010	1.425	0.710
Alpha-trim mean	α = 10%	**1.678**	0.604	2.691	0.558	1.019	1.428	0.665
	α = 15%	1.694	0.601	**2.642**	**0.409**	**0.928**	**1.214**	0.591
	α = 25%	1.735	0.615	2.759	0.428	1.020	1.492	0.628

**Table 4:** Finalized phase 1 experimental results on all seven participants using FAB and alpha-trim mean (α = 15%). The downward and upward arrows indicate better results when the respective smaller and larger metric values are obtained.

Participant #	MAE (lbs.) ↓	MSE (lbs.^2^) ↓	RMSE (lbs.) ↓	R^2^ ↑	MAPE ↓
1	1.694	3.834	1.958	0.755	0.009
2	0.601	0.735	0.857	0.949	0.0036
3	2.642	13.346	3.653	0.055	0.0175
4	0.409	0.3596	0.5997	0.981	0.0019
5	0.928	1.590	1.261	0.920	0.0085
6	1.214	1.916	1.384	0.897	0.0076
7	0.591	0.923	0.961	0.954	0.0037
**Average:**	**1.154**	**3.243**	**1.525**	**0.787**	**0.0074**

**Table 5:** Averaged results for phase 2 experiments with 37 participants (22 and 15 self-identifying males and females, respectively), using phase 1 finalized pipeline which includes using FAB addition, sample length of 100, and an alpha-trim mean with α = 15%.

# Participants	MAE (lbs.) ↓	MSE (lbs^2^) ↓	RMSE (lbs.) ↓	R^2^ ↑	MAPE ↓
37	1.009	2.483	1.369	0.785	0.0061

**Table 6:** Performance analysis of the proposed method with the existing deep-learning-based state-of-the-art method.

Author	Participants	Environmental Settings	Method	Sensor	Results
Kim et al. [10]	72	---	Modified MobileNetV2 using multi-task loss	Gilon, INC	MAE of 6.85 kg (15.10 lbs.)
**Proposed method**	**37**	**Less controlled**	**Individualized machine learning**	**Moticon** **OpenGO**	**1.009 lbs.**
	**7**	**Controlled**			**1.154 lbs.**

## Data Availability

Proposed dataset is private data and will be available on request for research purposes.
